# Wing pathology of white-nose syndrome in bats suggests life-threatening disruption of physiology

**DOI:** 10.1186/1741-7007-8-135

**Published:** 2010-11-11

**Authors:** Paul M Cryan, Carol Uphoff Meteyer, Justin G Boyles, David S Blehert

**Affiliations:** 1 United States Geological Survey, Fort Collins Science Center, Fort Collins, CO 80526, USA; 2 United States Geological Survey, National Wildlife Health Center, Madison, WI 53711, USA; 3 University of Pretoria, Department of Zoology and Entomology, Pretoria 0002, South Africa

## Abstract

White-nose syndrome (WNS) is causing unprecedented declines in several species of North American bats. The characteristic lesions of WNS are caused by the fungus *Geomyces destructans*, which erodes and replaces the living skin of bats while they hibernate. It is unknown how this infection kills the bats. We review here the unique physiological importance of wings to hibernating bats in relation to the damage caused by *G. destructans *and propose that mortality is caused by catastrophic disruption of wing-dependent physiological functions. Mechanisms of disease associated with *G. destructans *seem specific to hibernating bats and are most analogous to disease caused by chytrid fungus in amphibians.

## The emergence of a novel fungal pathogen

White-nose syndrome (WNS) was first observed in the United States during the winter of 2006-07 in caves and mines where bats hibernate (hibernacula), centered on a popular tourist cave in upstate New York [1]. During the three subsequent winters, large die-offs of bats were observed in zones radiating from that small area of New York through the karst regions of eleven states and two Canadian provinces (linear distances of approximately 1,300 km), resulting in the first sustained epizootic affecting bats in recorded history. Losses at affected hibernacula have exceeded 75% [1], and some winter colonies that were stable or increasing in number for decades have all but disappeared [2]. Biologists estimate that more than 1 million bats have died, which far exceeds the rate and magnitude of any previously known natural or anthropogenic mortality events in bats, and possibly in any mammalian group. All of the six species of cavernicolous hibernating bats that occur in WNS-affected areas have shown evidence of the disease and associated mortality [3,4]. It is assumed that as this disease spreads to new areas, each of the species of cave hibernating bats in those areas will also be at risk. The little brown bat (*Myotis lucifugus*), the most abundant species in the region currently affected by WNS, has experienced particularly dramatic population losses [5].

The characteristic lesions associated with WNS are caused by a newly described psychrophilic (cold-loving) fungus, *Geomyces destructans *[1,6,7], which also occurs on bats in Europe, but without the associated mortality [8,9]. Unlike other cutaneous fungal pathogens of endothermic animals, which cause superficial infections, *G. destructans *is capable of digesting, eroding and invading the skin of hibernating bats [7]. The white material on the muzzle of bats with WNS represents the prolific production of fungal conidia (spores) and is the most obvious field manifestation of WNS. Although the density of spore production around the muzzle is the most dramatic sign of infection, the skin of hibernating bat wings is the most significant target of *G. destructans *[7]. Bats have four to eight times more exposed skin membrane along their arms, digits and tail (hereafter 'wings') than on other parts of the body [10]. These disproportionately large areas of exposed skin play critical roles in homeostasis and thus in day-to-day survival. The apparent subtlety of pathology seen with the naked eye belies the prevalence, severity and extent of wing damage in WNS, and is likely to be one of the reasons for an underappreciation of *G. destructans *as a primary pathogen.

## The success of *G. destructans *relates to host physiology during hibernation

The natural cycle of hibernation has allowed *G. destructans *to become a highly successful emergent pathogen of bats. Hibernation, characterized by long cycles of deep torpor and intermittent arousal, is a strategy of endotherms for maximizing survival during seasonal periods of harsh conditions, food shortage and/or water limitations. During hibernation, immune function and metabolism are dramatically downregulated, and possibly even inhibited [11-14], with an accompanying drop in body temperature [15]. The hibernating temperature of bats is within the range for maximal growth of *G. destructans *(approximately 1 to 15°C) [1,6,7]. In addition to physiological changes, different species of bats have evolved different behavioral strategies to maximize survival during hibernation, such as selection of humid areas of hibernacula or dense clustering to conserve energy and decrease moisture loss [16-18]. These behaviors could further enhance fungal colonization, growth and conidial amplification by elevating humidity, as well as increasing infection rate and dispersal of *G. destructans *through increased contact with infected individuals. In addition, natural downregulation of immune function in hibernating species is likely to allow *G. destructans *to invade body tissues without confronting an immune response [14], making the hibernating bat a most accommodating host that provides nutrients, ideal environmental conditions and little or no resistance to an expanding infection.

## Pathology of *G. destructans *infection in the wings of hibernating bats

The US Geological Survey National Wildlife Health Center (NWHC) has been the primary diagnostic lab receiving bats for WNS assessment and defined the pathology that is diagnostic for this disease [7]. One of us (CUM) has carried out histologic evaluation on most of the bats submitted to the NWHC between October and June over the past three years (see Additional file 1). Of 285 bats examined at NWHC, 198 were histologically positive for WNS.

**Figure 1:**
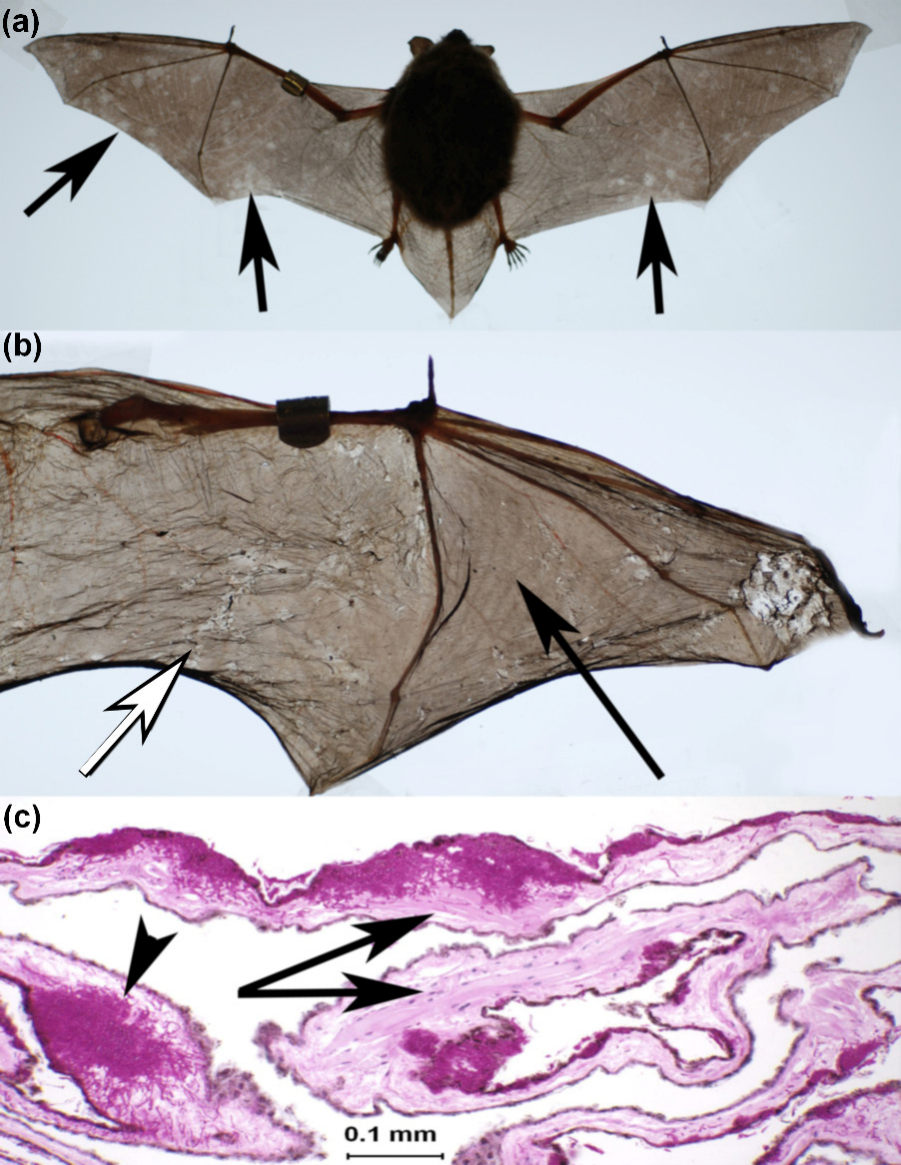
**The effects of *Geomyces destructans *infection on bat wings**. **(a) **Back-lit photograph of wings of a euthanized WNS-positive little brown bat (*Myotis lucifugus*) with subtle circular and irregular areas of pallor (arrows) in wing membrane. **(b) **Back-lit photograph of the wing of a euthanized little brown with significant visible pathology associated with WNS. Area of wing membrane with relatively normal tone and elasticity (black arrow), compared to an area that has lost tone, elasticity and surface sheen, with irregular pigmentation and areas of contraction (white arrow). **(c) **Periodic acid Schiff-stained, 4-μm histologic section of wing membrane prepared as previously described [7] from a *M. lucifugus *showing extensive fungal infection by *G. destructans*. Fungal hyphae replace muscle bundles (arrows); invasion can become transdermal with associated edema (arrowhead).

**Figure 2:**
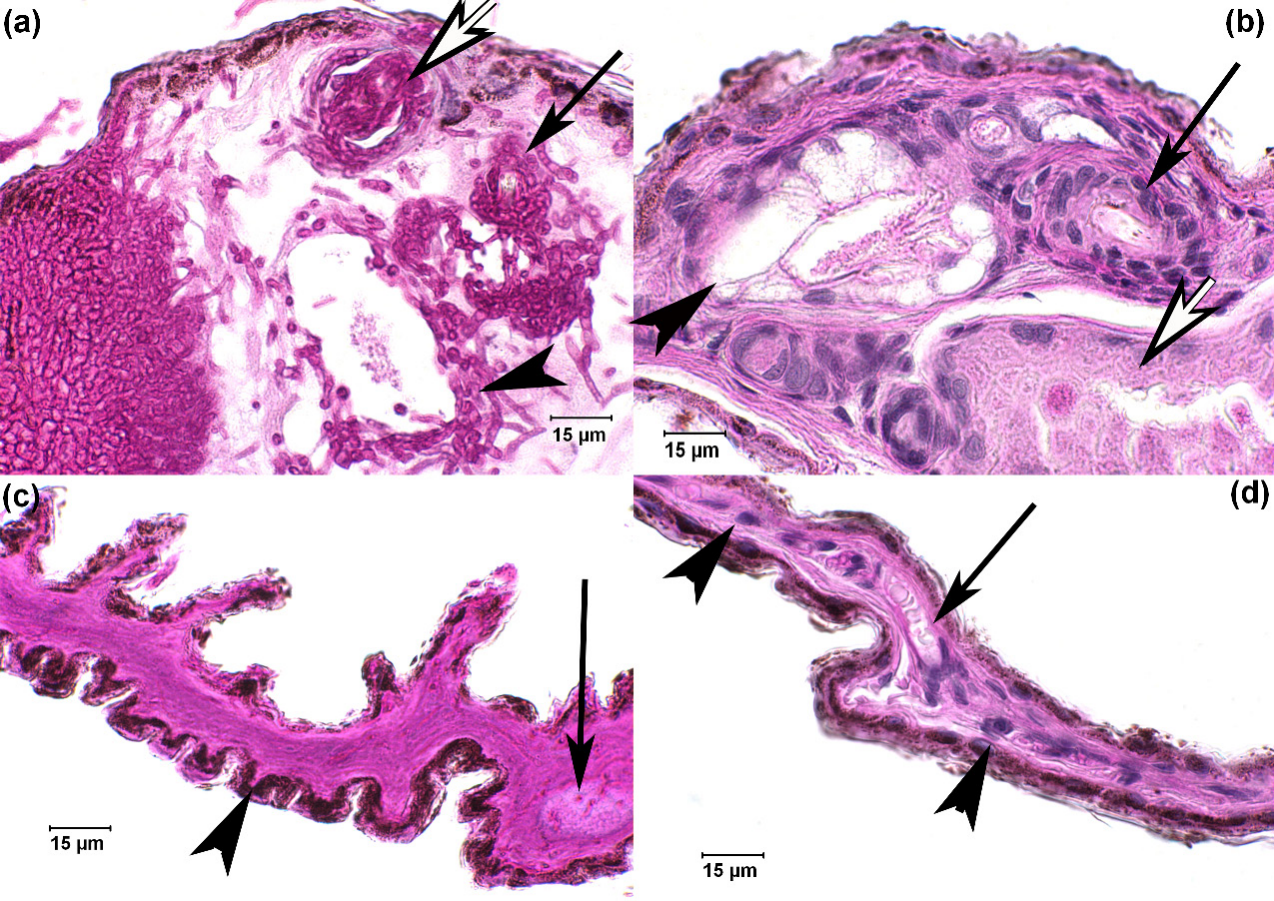
**Photomicrographs of periodic acid Schiff-stained 4-μm sections of wing membrane prepared as previously described **[7]**from a little brown bat (*Myotis lucifugus*) infected by *Geomyces destructans***. **(a) **Fungal hyphae penetrate and replace apocrine gland (white arrow), hair follicle (black arrow pointing to hair shaft), and sebaceous gland (arrowhead). **(b) **Normal pilosebaceous unit including the apocrine gland (white arrow), hair follicle (black arrow pointing to hair shaft) and sebaceous gland (arrowhead). **(c) **Infarcted region of wing membrane showing loss of all identifiable vital structures in the dermis, including blood vessels, connective tissue, muscle, elastin fibers and the large bands of connective tissue that traverse and stabilize wing membrane (arrow). No discernable cell structures or nuclei remain, the wing membrane is contracted and hypereosinophilic (intense red staining), and only residual pigment is present on the membrane surface (arrowhead). **(d) **Microscopic section of normal wing membrane with identifiable blood vessel containing circulating red blood cells (arrow) and nuclei of connective tissue cells (arrowheads).

The wing membranes of bats consist of two layers of epithelium separated by a thin layer of blood and lymphatic vessels, delicate nerves, muscles and specialized connective tissues [19,20]. The wings of winter-collected WNS bats often reveal subtle signs of infection when examined with the unaided eye (Figure 1a). Suppleness, elasticity and tone are obvious when a healthy wing is contracted or extended, or when the arm and digits are rotated. In WNS-affected bats, these characteristics of the wing membrane are compromised. Folded surfaces of severely affected wing membranes adhere to each other, tear easily [7], appear to lose tone, tensile strength and elasticity, and resemble crumpled tissue paper (Figure 1b). Microscopic examination of wings infected by *G. destructans *reveals a degree of damage that suggests functional impairment. Diagnostic features of WNS are fungal colonization of skin with epidermal erosions that are filled with fungal hyphae (Figures 1c and 2a) [7]. In addition to the cup-like erosions of the epidermis caused by *G. destructans*, fungal destruction of the apocrine glands, hair follicles and sebaceous glands that comprise the adnexa and deeper dermal invasion is common (Figure 2a). Connective tissue, blood and lymphatic vessels, glandular structures, and elastin and muscle fibers of normal wing tissue (Figure 2b,d) are replaced as *G. destructans *digests, uses and invades skin at the interface with the expanding colony (Figures 1c and 2a).

Infarction is the acute death of tissue due to loss of oxygen supply. Characteristic changes that define infarcted tissue were seen in regions of wing membrane that were distant from fungal invasion, including loss of all identifiable vital structures in the dermis, contraction of tissue and hypereosinophilia (an intense uniform red-staining of tissue) (Figure 2c). Other fungi have the ability to directly invade vessels, obstruct blood flow and cause infarction of tissue that depends on blood flow [21]. Although *G. destructans *is not vasculotropic - that is, it does not directly invade blood vessels - effacement of the vasculature caused by this fungus could have the same effect of terminating blood flow to a region. Inflammation in response to this winter fungal infection is usually lacking, as would be expected with the downregulation of immune function in mammals during hibernation.

Although *G. destructans *infections are limited to skin, and there is no consistent evidence that secondary bacterial infections are largely involved in the disease syndrome, the pathology caused by this fungus in wing structures suggests multiple life-threatening physiological effects on hibernating bats. Emaciation is a common finding in bats that have died from WNS; the link between emaciation and the cutaneous infection with *G. destructans *has not been elucidated, and we hypothesize that disruption of physiological homeostasis potentially caused by *G. destructans *is sufficient to result in emaciation and mortality.

## The role of wings in maintaining homeostasis: water balance and dehydration

Healthy wing membranes are critical for maintaining water balance in bats. Bats are especially susceptible to dehydration during winter hibernation [16,22,23]. The exposed wing membranes and large lungs of bats predispose them to evaporative water loss (EWL) [24,25], and losses from the skin alone can account for as much as 99% of total water loss in healthy hibernating bats [23,26]. EWL is inversely related to the humidity of surrounding air, and most hibernating bats select wintering sites with high humidity (typically 60 to 100% relative humidity) [16,23]). However, certain species of bats are, for unknown reasons, more susceptible to water loss and can lose water even while hibernating in very humid sites. For example, the small amount of surplus water produced as a byproduct of fat metabolism in solitarily hibernating *M. lucifugus *does not compensate for EWL except at levels of relative humidity greater than 99%, and this species regularly incurs water debt during bouts of winter torpor, even in hibernacula with near-saturated air [23].

Differences exist among species of hibernating bats in their selection of roost microclimates and susceptibility to EWL during hibernation [16,27,28]. It may not be a coincidence that species that have lower reported mortality or more variable declines due to WNS (*Myotis sodalis*, *Myotis leibii *and *Eptesicus fuscus*) are those that seem less susceptible to EWL, often select drier areas of hibernacula, and are rarely, if ever, seen covered with condensation during hibernation [16]. The three species most frequently diagnosed with WNS (*M. lucifugus*, *Myotis septentrionalis *and *Perimyotis subflavus*) are also those that consistently roost in the most humid parts of hibernacula and are often observed with condensation on their fur [16], suggesting that these species are more susceptible to EWL and have evolved compensatory behavioral strategies, such as roost selection or hibernation in tight clusters. Paradoxically, these behavioral adaptations may put the latter species at greater risk of infection with *G. destructans *and subsequently at greater risk of the dehydration that could result from fungal damage to wings.

Infection with *G. destructans *can lead to extensive loss of dermal integrity (Figures 1c and 2a). It is logical to infer that any regulation of fluid balance that requires intact skin would also be lost in WNS-infected bats. On the basis of the pathology associated with WNS, we hypothesize that *G. destructans *impairs skin-mediated fluid regulation to the extent that behavioral strategies used by hibernating bats to restore water balance, such as roost selection, licking condensation from fur and short flights to drink surface water [16], may be inadequate to prevent excessive water loss and clinical dehydration. Necropsy findings from bats with severe *G. destructans *infections support dehydration as a contributory factor to mortality. For example, pectoral muscles of *M. lucifugus *that died with WNS were usually congested and so adherent to a gloved finger (a qualitative indicator of antemortem dehydration) that carcasses could be lifted off the necropsy table.

It is also possible, as in fungal infections of invertebrates [29], that epidermal fungal growth may increase the evaporative surface area of bat wings or wick water from the wing membrane at points of exuberant fungal proliferation, such as skin glands. Aggressive invasion by *G. destructans *also destroys hair follicles, and sebaceous and apocrine glands (Figure 2a,b), and thus eliminates protective secretions in regions of infected skin [20,30-32]. These secretions moisturize and waterproof skin [32], may provide a protective barrier against harmful microorganisms, and are likely to supply nutrients to symbiotic microorganisms [31].

## Links between dehydration and depletion of fat stores

Fat (energy) available to hibernating bats is accumulated in the weeks before winter when insect prey is available. During most of the hibernation period, a bat expends relatively little energy by maintaining its core body temperature close to ambient air temperature, usually about 0 to 10°C [17,33,34]. Much of the energy expended during hibernation is used to fuel brief, periodic arousals from torpor when body temperature is raised to the level of their non-hibernating warm-blooded (euthermic) state (35 to 39°C) [34,35]. Although arousals from torpor are a major factor influencing winter energy expenditure and thus over-winter survival, surprisingly little is known about what triggers them [23]. Arousals are thought to be necessary for maintaining homeostasis (for example, restoring neural and muscular function, excreting waste and replenishing water and energy stores) [35], and one of the long-standing hypotheses for explaining the frequency of arousals in healthy bats is the need for hibernating bats to drink and restore water balance [16,23,33,36]. Although a prevailing hypothesis is that the symptomatic daytime flight of WNS-affected bats outside caves and mines in mid-winter is the result of starving bats emerging from hibernation sites in a last-ditch effort to find insect prey [4], there is sufficient evidence to suggest that thirst may be driving these arousals. We hypothesize that wing damage caused by *G. destructans *could sufficiently disrupt water balance to trigger frequent thirst-associated arousals with excessive winter flight, and subsequent premature depletion of fat stores resulting in the emaciation associated with WNS. This hypothesis inextricably links water balance and depletion of stored energy during hibernation and places thirst as the potential driving stimulus for abnormal arousals. Anecdotally, bats at hibernacula affected by WNS are sometimes seen flying over and drinking from water surfaces or eating snow (A Hicks, personal communication), highlighting the plausibility of the dehydration hypothesis.

## Disruption of circulation and cutaneous respiration by *G. destructans*

In addition to the potential for wing damage caused by *G. destructans *to negatively influence water balance, and consequently energy consumption, infection with the fungus may also disrupt blood circulation and cutaneous respiration. Vessels in the thin wing membranes of bats are easily observed through the single layer of epidermis, and physiologists interested in mammalian circulation have been studying the vasculature of bat wings for over a century [20,37]. General vascular structure in the bat wing is similar to that in the skin of other mammals, with arterioles, veins and dense capillary beds that supply nutrients and remove metabolic waste. In addition, the wing veins of bats produce rhythmic peristaltic contractions that help move blood toward the heart during flight and when roosting upside-down, precapillary sphincters that regulate blood pressure in capillary beds, and venous anastomoses that can shunt blood away from the capillary beds by diverting it directly into the venous system from arteries [37,38]. Wing vessels also serve as reservoirs that regulate blood pressure using specialized adaptations that allow bats to quickly transition from inert, upside-down postures to active flight [37,38]. The histopathology does not indicate that *G. destructans *is vasculotropic, but fungal erosion and progressive destruction of all components of skin, including the vessels, would alter the physical relationships that normally exist between the environment, epidermis, connective tissue and regional vasculature. Damage could obstruct blood flow directly or through increases in pressure and retrograde dilation of capillaries, arterioles, veins, and lymphatic vessels. Although not a defining characteristic of WNS pathology, the presence of wing membrane infarction (Figure 2c), usually the result of arteriolar occlusion, lends observational support to the hypothesis that significant circulatory disturbance is even more extensive than the necrosis caused by direct erosion and invasion of the tissues by fungal hyphae.

As red blood cells are transported through the circulatory system from the lungs to distant tissues, including a bat's wings (Figure 2d), they provide oxygen. Circulation also removes metabolic byproducts such as carbon dioxide (CO_2_). However, because the blood-gas barrier of the wing membrane is so thin, substantial gas exchange also occurs between the wing and the surrounding air directly through transpiration. Studies have shown that bat wings release remarkable amounts of CO_2 _in warm temperatures (for example, 10% of total gas exchange in *E. fuscus *at 35°C [39]), and that the wings of some species take up similar amounts of O_2 _(for example, 10% of total gas exchange in *Epomophorus wahlbergi *at 33°C [19]). Though rates of cutaneous gas exchange in bats decrease with metabolic downregulation during torpor, such passive gas exchange in hibernating bats may be especially important during extended periods of hibernation when respiration rates are extremely low [19,39]. Passive gas exchange through the wings of hibernating *M. lucifugus *and *E. fuscus *has been documented during the physiological periods of hibernation-induced apnea when the frequency of respirations drops dramatically [40-42]. Recent evidence suggests that passive gas exchange across wing surfaces could occur during hibernation, even when the wings are folded [19]. The damage to gas-permeable wing membranes and the associated vasculature by *G. destructans *suggests disruption of effective transpiration across the wing surfaces and subsequent compromise of total respiratory gas exchange during hibernation. Lower passive gas exchange across wing surfaces could potentially trigger compensatory respiration through the lungs, leading to increased pulmonary evaporative water loss.

## Disruption of thermoregulation by *G. destructans*

It has been hypothesized that infection by *G. destructans *alters the normal arousal cycles of hibernating bats, particularly by increasing arousal frequency and/or duration [43]. Increased heat-generation demands during these abnormal arousals may also contribute to premature depletion of energy reserves, emaciation and death. During arousals from hibernation, a bat must produce enough metabolic heat to raise its body temperature about 20 to 35°C over the course of minutes to hours [33]. It is a considerable challenge to metabolically heat a small body with a large skin surface area while hanging upside-down inside a cold, dark and damp underground site, and may be a losing battle for bats with wings infected by *G. destructans*.

The epidermis and circulatory system of bat wings contribute to the regulation of core body temperature by heat retention or transfer at the epithelial surface [10,24,37,38]. Destruction of the epithelial barrier in regions of skin infected by *G. destructans *is likely to increase the rate of heat flux out of the body. Blood of an arousing bat is warmed as it circulates through the body core with the aid of highly vascularized and thermogenic brown adipose tissue [37,38]. In healthy bats, the flow of warmed blood is restricted in peripheral tissues during arousal [35], thus reducing heat loss to ambient air at the wing surfaces. If blood vessels or anastomoses involved in restriction of peripheral blood flow are damaged, or the epidermal barrier is breached, warmed blood could quickly lose heat through the wings, placing a greater energetic cost on re-warming during arousals and more rapidly depleting limited fat reserves. Wing damage caused by *G. destructans *could initiate an unsustainable cycle of energy loss.

## Fungal impairment of flight

An obvious effect of wing damage is the alteration of the aerodynamic properties of the wing [2]. Researchers working in WNS-affected regions during spring and summer have reported serious wing damage on bats, indicating that infection by *G. destructans *may compromise the health and reproductive success of survivors during the warmer months when they are active, primarily by decreasing flight efficiency [2]. However, almost all of the documented mortality associated with WNS has been during hibernation. Hibernating bats arouse from torpor and fly during mid-winter to drink, change roost locations and occasionally forage [44]. These behaviors become abnormally frequent in bats affected by WNS and infected bats have been observed to wing-walk on snow, unable to fly. Mechanical impairment of flight is a likely result of wing damage associated with *G. destructans*. Bat wings are highly innervated [37], and fungal penetration or biochemical alteration of innervated tissues in the wing could destroy nerves and touch receptors necessary for effective locomotion. Touch-sensitive hair-cell receptors found throughout the wings of bats are thought to sense airflow across wing surfaces, and probably play a critical role in controlling flight [45,46]. Touch receptors associated with pilosebaceous units infected with *G. destructans *are likely to be destroyed as these structures are invaded by fungus. Elastin, fibrin and collagen degeneration, necrosis of localized muscle, and damage to large suspensory connective tissue bands that traverse the wing (Figure 2c) could also disrupt flight control and stabilization of the wing.

## Comparison with other cutaneous fungal pathogens

Cutaneous fungal pathogens other than *G. destructans *that infect invertebrates interfere with water balance of the host. Laboratory experiments reveal that fungal infections cause death by dehydration in dog ticks (*Dermacentor variabilis*), even at higher levels of humidity (greater than 90% relative humidity at 25°C) than are typically sustained under natural conditions [29]. In certain insects, symbiotic fungi in the glands of normal cuticle help maintain homeostasis and prevent infection by pathogenic conidial fungi; without these symbionts, pathogenic fungi colonize the cuticle and subsequently cause death by dehydration [29].

Although *G. destructans *infection is limited to skin, its severe invasion and replacement of skin structures is not characteristic of typical dermatophytes such as *Microsporum gypseum*, *Trichophyton rubrum *and *Geomyces pannorum*. Dermatophytes of mammals typically colonize the superficial epidermis, hair and nails and do not invade living tissue [47]. The ability of *G. destructans *to invade the wing skin of hibernating bats is unlike that of any known cutaneous fungal pathogens in terrestrial mammals. As discussed in this article, we propose that damage to the bat wing, a physiologically dynamic membrane, brought about by *G. destructans *is sufficient to directly cause mortality.

**Figure 3:**
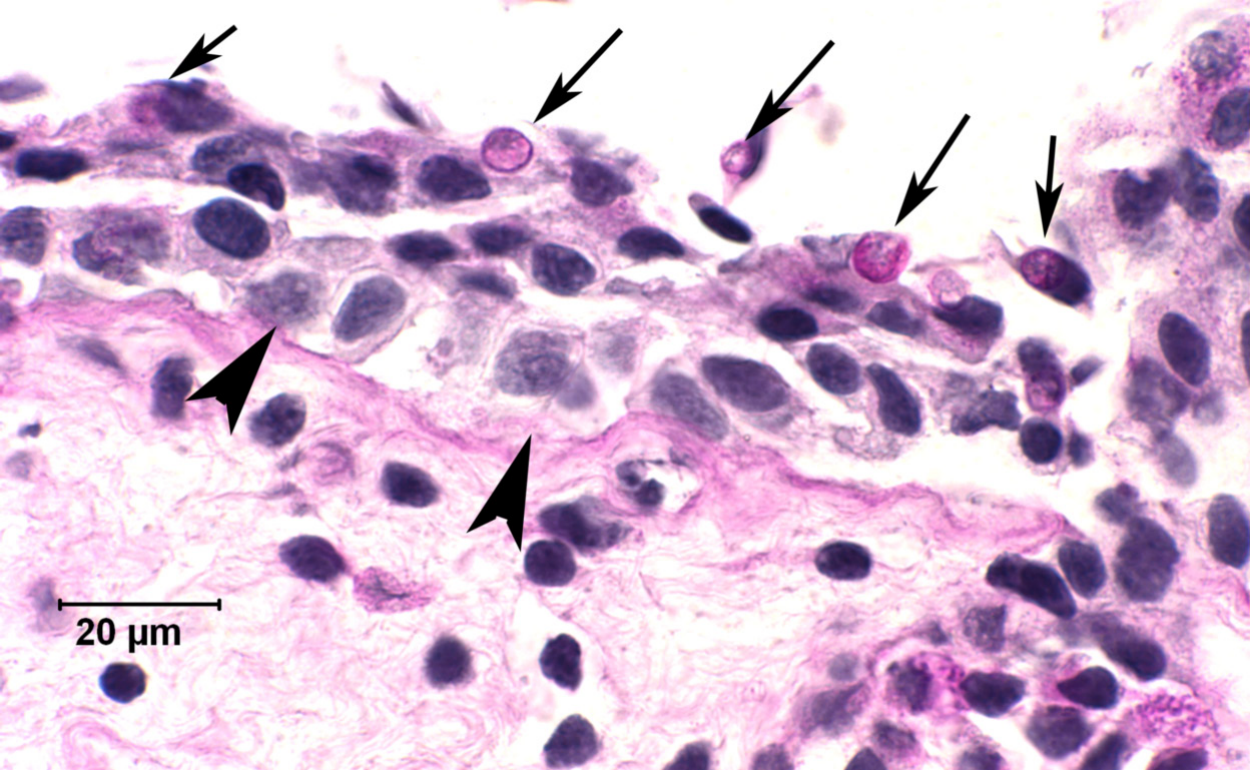
**Periodic acid Schiff-stained, 4-μm histologic section of skin from a lowland leopard frog (*Rana yavapaiensis*) infected with the chytrid fungus *Batrachochytrium dendrobatidis***. *B. dendrobatidis *(arrows) has colonized the superficial epidermal layer of frog skin. Physiological response to fungal infection includes thickening of the keratin layer (most lost in processing) and increased cells in the epidermis (cells between arrows and arrow heads), but there is no inflammation.

The potential homeostatic imbalance associated with the damage *G. destructans *causes in bat wings warrants comparison to the electrolyte imbalance that occurs in amphibians infected by chytrid fungus (*Batrachochytrium dendrobatidis*) [48]. Recent studies demonstrated that infection by *B. dendrobatidis *impairs the ability of frog skin to regulate hydration and homeostasis, causing electrolyte imbalance and ultimately cardiac arrest [49]. Like WNS in hibernating bats, chytridiomycosis has caused precipitous declines among multiple species of wild amphibians. Additional similarities between skin infections of hibernating bats by *G. destructans *and of amphibians by *B. dendrobatidis *include the critical role the skin plays in the physiology of both hosts, as well as a lack of host inflammatory response to both cutaneous pathogens. The lack of inflammation in frogs is due to the superficial nature of infection. The lack of inflammation in bats is likely to be the result of natural downregulation of the mammalian immune system during hibernation [11-14]. A dramatic difference between these host-pathogen relationships is the limited nature of epidermal invasion by *B. dendrobatidis *in amphibians (Figure 3) compared with the severe erosion, invasion and destruction of living tissues by *G. destructans *(Figures 1c and 2a).

Despite the relatively minor visible changes associated with *B. dendrobatidis *infections, it is still a lethal physiological pathogen because of the role that the amphibian skin plays in the regulation of hydration and blood chemistry. We suggest that a similar, but less subtle, perturbation could be occurring in the wing membranes of bats with WNS. Damage to bat wings caused by *G. destructans *is often more extensive than can be appreciated with the naked eye. It took researchers decades to establish the causal link between skin infection by *B. dendrobatidis *and mortality in amphibians. A contributing factor to this delay was the challenge of demonstrating the potential significance of what appeared to be a superficial infection, and then documenting the magnitude of its physiological consequences. In addition, this novel fungal pathogen of amphibians belonged to a genus that was previously known only as a saprophyte that did not infect vertebrates - it was a new disease paradigm. Infection of bat wings by *G. destructans*, also a member of a genus typically defined as saprophytes, may similarly represent a completely new disease paradigm for mammals.

Answers to the relationship between skin infection by *G. destructans *and bat mortality may be close to the surface. On the basis of available evidence and logical arguments, we have presented here numerous testable hypotheses for linking fungal infection of bat wings to WNS mortality. In summary, we hypothesize that *G. destructans *may cause unsustainable dehydration in water-dependent bats, trigger thirst-associated arousals, cause significant circulatory and thermoregulatory disturbance, disrupt respiratory gas exchange and destroy wing structures necessary for flight control. A promising approach to a better understanding of WNS mortality might be to compare the North American disease to infection of bats by *G. destructans *in Europe, where associated mortality is not apparent. If explanatory differences are not found between continents in the pathogen (for example, differences in fungal virulence) or environment (for example, the duration and severity of winters [9]), then some of the host physiological or behavioral mechanisms we have outlined may help explain mortality in North American bats. Physiological differences between European and North American hibernating bats are unknown, but might include differences in host immune response [8,9], differences in rates of cutaneous water loss (for example, differences in skin secretions, gland prevalence and structure), differences in the symbiotic organisms supported [9], or differences in tolerance of dehydration or other physiological stress during hibernation. Host behavioral differences linked to physiology and potentially influencing the susceptibility of bats in different continents might include the size of groups formed [9], the humidity and temperature ranges chosen for hibernation, typical activity levels (for example, foraging or drinking) during hibernation, or stereotyped responses to 'disturbance'. We urge further research into the physiological consequences of skin infection by *G. destructans *and its impact on survival - with more than 150 years of detailed knowledge about the anatomy and physiology of bat wings, understanding the effect of WNS on bat wings seems tractable with available methods and expertise.

## Authors' contributions

PC and CM co-developed the conceptual framework of this synthesis and drafted the manuscript. DB and JB played substantial roles in expanding and improving concepts related to mycotic diseases and bat physiology, respectively, and drafting the manuscript. All authors read and approved the final manuscript.

## Supplementary Material

Additional file 1 **Methods**. A Word document containing details of Methods. Click here for file
