# Supplementary material for: Wing pathology of white-nose syndrome in bats suggests life-threatening disruption of physiology
Source: BMC Biol. 2010 Nov 11;8:135. doi: 10.1186/1741-7007-8-135 (PMC2984388; doi:10.1186/1741-7007-8-135)
Supplement: Additional file 1 — Methods. A Word document containing details of Methods. [file 1741-7007-8-135-S1.DOC]

**Additional file 1**

# Methods

Ethics Statement: All procedures for sampling bats that generated data used in this review were approved by Institutional Animal Care and Use Committees of the US Geological Survey following the American Veterinary Medical Association's Guidelines on Euthanasia and the Guidelines of the American Society of Mammalogists for the Use of Wild Mammals in Research and by the USGS National Wildlife Health Center Animal Care and Use document #EP081124-A1.

Bats with or without evidence of WNS from within or around hibernation sites were collected dead or euthanized, then chilled and shipped overnight to the NWHC for necropsy and diagnostic testing, with consistent sampling and microscopic examination of wing membrane (Figure 2a). Collection of bats was opportunistic, rather than targeted, yet consistent pathological findings were observed in WNS bats sampled across morthan 1,300 km of the eastern United States. Wing membranes were prepared for microscopic examination as previously described [7]. Briefly, back-lit photographs of wings were taken using a photographic light box, and then samples of membrane were cut and rolled onto 0.2 x 3.0 cm wooden dowels before being placed in 10% neutral buffered formalin. After a minimum 48-hour formalin fixation, rolls of wing membrane were removed from dowels, cross-sectioned, and resulting spiral sections were embedded in paraffin with their cut surfaces down to maximize the length of continuous wing membrane available for histologic examination. The paraffin embedded spirals of tissue were then cross-sectioned at 4 μm, mounted on glass slides, and stained with Periodic acid Schiff to optimize microscopic observation of fungal hyphae (Figure 2b). A qualitative assessment of antemortem dehydration was also made by evaluating degree of pectoral muscle adhesion to a gloved finger. Similar shipping, necropsy, and histology methods were applied to the lowland leopard frog (*Rana yavapaiensis*) submitted to the NWHC and subsequently diagnosed with skin infection by *B. dendrobatidis* (Figure 3)*.*
